# Feasibility, Acceptability, and Potential Impact of a Novel mHealth App for Smokers Ambivalent About Quitting: Randomized Pilot Study

**DOI:** 10.2196/46155

**Published:** 2023-06-28

**Authors:** Jennifer B McClure, Jaimee L Heffner, Chloe Krakauer, Sophia Mun, Predrag Klasnja, Sheryl L Catz

**Affiliations:** 1 Kaiser Permanente Washington Health Research Institute Seattle, WA United States; 2 Kaiser Permanente Bernard J Tyson School of Medicine Pasadena, CA United States; 3 Cancer Prevention Program Public Health Sciences Division Fred Hutchinson Cancer Center Seattle, WA United States; 4 School of Information University of Michigan Ann Arbor, MI United States; 5 Betty Irene Moore School of Nursing University of California, Davis Sacramento, CA United States

**Keywords:** ambivalence, app, digital health intervention, mHealth intervention, mHealth, motivation, nicotine, smoking, smoking cessation, tobacco

## Abstract

**Background:**

Most smokers are ambivalent about quitting—they want to quit someday, but not now. Interventions are needed that can engage ambivalent smokers, build their motivation for quitting, and support future quit attempts. Mobile health (mHealth) apps offer a cost-effective platform for such interventions, but research is needed to inform their optimal design and assess their acceptability, feasibility, and potential effectiveness.

**Objective:**

This study aims to assess the feasibility, acceptability, and potential impact of a novel mHealth app for smokers who want to quit smoking someday but are ambivalent about quitting in the near term.

**Methods:**

We enrolled adults across the United States who smoked more than 10 cigarettes a day and were ambivalent about quitting (n=60). Participants were randomly assigned to 1 of 2 versions of the GEMS app: standard care (SC) versus enhanced care (EC). Both had a similar design and identical evidence-based, best-practice smoking cessation advice and resources, including the ability to earn free nicotine patches. EC also included a series of exercises called experiments designed to help ambivalent smokers clarify their goals, strengthen their motivation, and learn important behavioral skills for changing smoking behavior without making a commitment to quit. Outcomes were analyzed using automated app data and self-reported surveys at 1 and 3 months post enrollment.

**Results:**

Participants who installed the app (57/60, 95%) were largely female, White, socioeconomically disadvantaged, and highly nicotine dependent. As expected, key outcomes trended in favor of the EC group. Compared to SC users, EC participants had greater engagement (mean sessions 19.9 for EC vs 7.3 for SC). An intentional quit attempt was reported by 39.3% (11/28) of EC users and 37.9% (11/29) of SC users. Seven-day point prevalence smoking abstinence at the 3-month follow-up was reported by 14.7% (4/28) of EC users and 6.9% (2/29) of SC users. Among participants who earned a free trial of nicotine replacement therapy based on their app usage, 36.4% (8/22) of EC participants and 11.1% (2/18) of SC participants requested the treatment. A total of 17.9% (5/28) of EC and 3.4% (1/29) of SC participants used an in-app feature to access a free tobacco quitline. Other metrics were also promising. EC participants completed an average of 6.9 (SD 3.1) out of 9 experiments. Median helpfulness ratings for completed experiments ranged from 3 to 4 on a 5-point scale. Finally, satisfaction with both app versions was very good (mean 4.1 on a 5-point Likert scale) and 95.3% (41/43) of all respondents would recommend their app version to others.

**Conclusions:**

Ambivalent smokers were receptive to the app-based intervention, but the EC version, which combined best-practice cessation advice with self-paced, experiential exercises, was associated with greater use and evidence of behavior change. Further development and evaluation of the EC program is warranted.

**Trial Registration:**

ClinicalTrials.gov NCT04560868; https://clinicaltrials.gov/ct2/show/NCT04560868

## Introduction

Tobacco use is responsible for over 8 million deaths per year worldwide [1]. The health risks of smoking are widely known and well documented, but the addictive properties of nicotine make quitting smoking difficult [2]. This explains why the majority of people who smoke want to quit someday but are not yet ready to commit to giving up tobacco anytime soon. This finding holds true across time, cultures, and countries [3-7].

When people are ready to quit smoking, effective, evidence-based treatment is widely available. This includes a combination of quit advice, supportive counseling, and pharmacotherapy [8,9]. However, to meaningfully reduce population smoking rates worldwide, a broader public health approach is required. Specifically, interventions are needed for those who want to quit smoking someday but are not yet ready to commit to change or to take action. These people are typically not eligible for cessation treatments (such as counseling or pharmacotherapy), which are largely limited to those who are ready to stop smoking.

While some may assume that people who are ambivalent about quitting smoking are not interested in an intervention, research has shown these individuals will enroll in smoking-focused intervention trials [10-14]. This illustrates that they are open to receiving information and assistance, despite their ambivalence about quitting smoking in the near term. A recent meta-analysis of 22 studies also found that interventions in this population can be as effective as interventions targeted to people who are ready to quit; however, the cost of intervention is substantially higher [13]. For example, the pooled cost per quit among smokers who are not yet ready to quit was US $19,510 for pharmacological interventions, US $11,416 for behavioral interventions, and US $14,662 for combined pharmacological and behavioral interventions compared to estimated costs per quit among smokers who were ready to quit, which ranged from US $1807 to US $3326 for behavioral interventions and US $2655 to US $3108 for combination therapy [13]. This cost is a prohibitive barrier for many health care providers, health care systems, and public health agencies, which is why provision of smoking cessation services is typically limited to people who are ready to quit and not offered to those who are ambivalent about quitting. However, this means the majority of smokers are excluded from intervention opportunities, even though they could benefit from them. To further reduce smoking prevalence, new intervention strategies that are both effective and cost-effective are needed for people who are ambivalent about quitting smoking.

We contend that mobile health (mHealth) apps offer a promising platform for intervening with people who are ambivalent about quitting. App-based interventions can have wide population-level reach with relatively little per-person intervention cost. From a user standpoint, they are also convenient and accessible. These benefits have helped drive the ballooning demand for and availability of digital health therapeutics and mHealth apps in recent years [15,16]. Yet, to our knowledge, there are no publicly available mHealth apps at this time that are designed specifically for smokers who are not ready to quit smoking.

It remains an open question whether ambivalent smokers would use an app-based smoking intervention if they are not ready to quit. Although, they would be interested in using an app to help them change their smoking behavior [17-19], especially if the app is responsive to their goals, such as reducing how much they smoke, and if they are not asked to commit to quitting. To date, only 1 published trial has evaluated app-delivered intervention in this population [20]. This study tested a comprehensive intervention that combined daily text messages (motivational support and quizzes) with financial incentives, encouragement to use 1 or more self-selected relaxation and distraction apps, motivational phone support from a tobacco treatment specialist, and precessation use of nicotine replacement therapy (NRT). While the 3-week intervention significantly enhanced quit rates at 6-month follow-up, more research is needed to confirm ambivalent smokers’ interest in using app-based smoking interventions and to inform their optimal design.

The primary objective of this randomized pilot study was to evaluate the feasibility and acceptability of using an mHealth app called GEMS to motivate and support smoking behavior change among smokers who want to quit smoking someday but have not yet. Two versions of GEMS were evaluated, each using a similar, but not identical, graphical user interface and content. The standard care (SC) version offered best-practice cognitive behavioral advice and other resources recommended for people who are ready to quit smoking, including access to cessation counseling and pharmacotherapy. The enhanced care (EC) version included this same content plus a series of specific cognitive and behavioral exercises designed to build motivation and enhance self-efficacy to reduce smoking or quit, and to promote quit attempts and cessation. We hypothesized that participants would use both versions of the app, but the EC version would have greater program use and, in turn, better support change in motivation, self-efficacy, and smoking behavior. However, this pilot study was not powered to detect statistically significant differences in cognitive or behavioral outcomes between the 2 app versions. Instead, findings will inform the need for further evaluation of the GEMS app and could inform the design of similar app-based interventions targeting smokers who are ambivalent about quitting smoking.

## Methods

### Ethics Approval

All research activities were conducted at the Kaiser Permanente Washington (KPWA) Health Research Institute and approved by the KPWA Institutional Review Board (#2020). Data were collected between December 2020 and October 2021. The study is registered with ClinicalTrials.gov (NCT04560868).

### Study Design

The study used a parallel, 2-arm design. Participants were randomly assigned to the SC or EC version of the app using an automated, block-stratified randomization scheme (≥15 cigarettes per day vs 10-14 cigarettes). This scheme ensured balanced representation of lighter versus heavier smokers between intervention arms since this could impact users’ motivation or ability to change their smoking behavior. Participants were followed for 3 months post enrollment and completed self-report surveys at 1 and 3 months post enrollment. Consistent with the purpose of a pilot trial [21], the goal of this work is to provide proof of concept for the app’s feasibility and acceptability.

### Recruitment, Eligibility, and Randomization

Participants were recruited through social media ads and screened for eligibility by phone. Individuals were eligible if they met the following criteria: 18 years of age or older; could read and speak in English; smoked at least 100 lifetime cigarettes; smoked in the past week; smoked at least ten cigarettes a day; wanted to quit smoking someday, but not in the next month; reported daily smartphone use; self-reported they could read text on their phone screen; were willing to download and install the app; and used either an Android or Apple smartphone. Individuals were excluded if they reported a lifetime history of dementia, manic depression (bipolar disorder), schizophrenia, contraindications for NRT use (pregnant, nursing, recent heart attack, or uncontrolled arrhythmia); another member of their household was already enrolled in the study; or if we were unable to verify the validity of their phone number or email address.

After completing the baseline survey and being randomized, participants were instructed on how to install the app and access their assigned intervention (EC vs SC). Those who failed to install the app within 3 days were offered assistance. Those who failed to install the app during the 3-month study period were excluded from the analytic sample, ensuring evaluative feedback was only collected from individuals who installed the app.

### Intervention Design, Content, and Functionality

#### General Overview

Both app versions were called GEMS. The name was chosen based on user feedback. Ambivalent smokers liked the name because it did not suggest the app was smoking related, making it more confidential and more appealing than a name implying the app was focused on smoking cessation.

After installing the app and setting up a user account, participants viewed a welcome screen, which explained the program’s purpose and a brief tutorial and orientation to the app’s features. Content could then be accessed ad-lib until completion of the 3-month follow-up survey, which concluded study participation. At this point, the study team remotely deactivated app access and ceased app usage tracking. Both versions of the app (SC and EC) had a similar design and identical content, except for the addition of the novel experiments in the EC version. This design meant that both groups received an active intervention and provided data useful to assessing the concept of offering an app-based intervention to people who were ambivalent about quitting, while also allowing us to assess differences that might be attributed to the additional features in the EC version. Shared and unique features of each app version are described below.

#### SC Content and Features Common to Both App Versions

The SC content was based on evidence-based treatment grounded in the US Public Health Service Guidelines for Treatment of Nicotine Dependence [9] and standard cognitive behavioral therapy for smoking cessation [22], with additional content and features informed by user-centered design work conducted by our team [17,18,23]. Messaging acknowledged users were not ready to stop smoking, but content focused on how to stop smoking, as per usual care treatment for smoking cessation. For example, the main feature of this program version was a Quit Guide that included advice on how to quit smoking; didactic information (eg, what is nicotine withdrawal, how does pharmacotherapy work); and a 6-step guide on how to quit (eg, how to choose and use stop-smoking medicines, how to set a quit date, how to prepare for your quit date, what to do on your quit date, and how to stay the course and prevent relapse). Participants could also call a nationwide tobacco quitline from within the app to enroll in free counseling available to all US residents. Other content included a calculator for estimating how much money could be saved by quitting smoking, a daily cigarette tracker, and 2 sets of narrative peer advice presented through short testimonials: 1 set offering motivational encouragement for quitting smoking and 1 modeling how to talk back to common excuses people give for smoking or not quitting. Finally, participants could keep notes on their quitting progress using an in-app journal.

Participants earned badge rewards (gems—hence the app name) for using app features (eg, saving calculator, daily cigarette tracker) and for viewing psychoeducational content (eg, each Quit Guide step). Participants were asked to actively indicate when they read key content by clicking a “Mark as Read” button on each page. Participants using the SC version of the app could earn up to 10 usage badges. After 6 badges were earned, users in both groups could request a free 2-week trial of NRT to help them stop smoking.

#### Experimental App Content and Unique Features

The EC app version mirrored the SC’s design, content, and functionality with 3 key exceptions. First, the home page of the EC version included, as the main content, a series of 9 cognitive and behavioral exercises called experiments. Each experiment was designed to help users clarify their values, build and strengthen their motivation for reducing or quitting smoking, and enhance their self-efficacy for changing smoking behavior by learning specific skills that could help them manage cravings and resist the urge to smoke (Table 1). Unless EC users opted to block text reminders, they also received reminder prompts to initiate or complete experiments. Second, in the EC version, the Quit Guide was in the resource toolbox, accessible from the home page, but it was less prominent than in the SC version, where it occupied the home page. Third, EC participants could earn up to 19 total badges: 9 for completing each of the experiments and 10 for viewing the program content common with the SC version.

**Figure 1:**
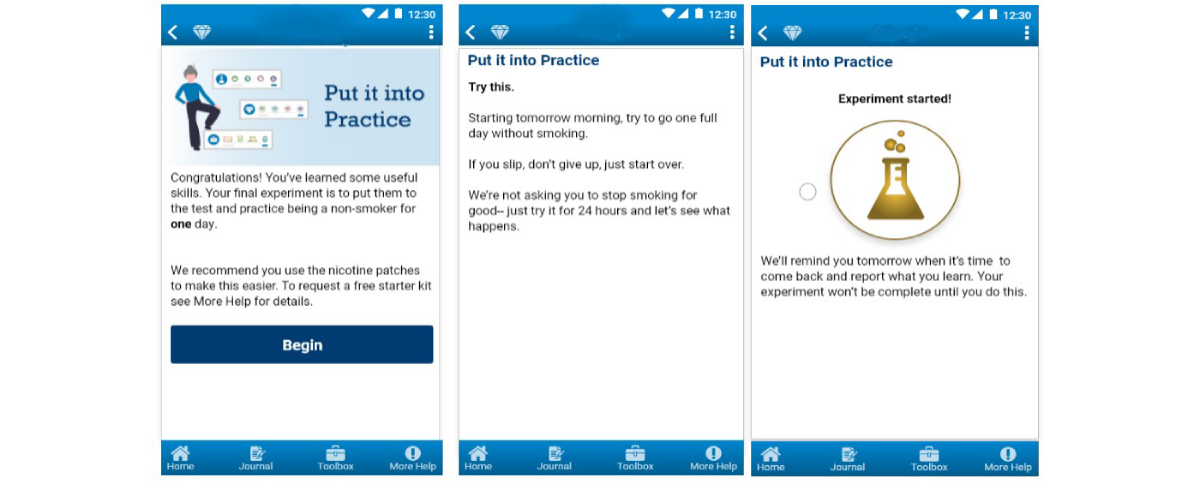
Example of experiment setup.

The theoretical rationale for the experiments, an overview of their design and flow, and preliminary formative research testing their acceptability and potential impact with smokers ambivalent about quitting have been previously reported [19]. Briefly, the EC intervention is grounded in empirically validated recommendations for treating nicotine dependence [9] and several complementary motivation and behavior change theories (eg, the PRIME theory of motivation [24,25], cognitive behavioral therapy, acceptance and commitment therapy, and social cognitive theory [26-28]). The experiments’ design was further informed by Fogg’s model of persuasive design [29], which suggests that when people have low motivation for change (as is the case with smokers ambivalent about quitting), the behaviors they are expected to engage in should be simple (ie, require low ability) and coupled with extrinsic triggers to prompt engagement (ie, reminder prompts). The specific behavioral goals and skills targeted in each experiment are summarized in Table 1 and an example is depicted in Figure 1.

With the exception of the first experiment, which could be completed in a few minutes, each exercise was designed to be practiced for a 24-hour period, after which participants were prompted to return and report what they learned by answering a brief series of reflective questions. Emphasis was placed on trying and learning from each exercise, as opposed to mastery or success, to avoid creating a sense of failure if participants did not complete or master the experiment. The experiments also built on one another, so lessons and skills learned in earlier experiments were designed to support success with later experiments.

To encourage sequential completion and forward progress, each experiment unlocked after completion of the previous experiment. If an experiment was started but not completed, the next experiment automatically unlocked after several days.

**Table 1:** Enhanced care experiments’ targeted skills and goals.

Experiment	Targeted skills and goals
One	Clarify personal values and health goals. Explore how smoking fits with these.
Two	Identify personal reasons for quitting. Build motivation for change.
Three	Identify high-risk situations for smoking. Inform future problem-solving and preparation for quitting.
Four	Learn deep breathing as a tool for stress reduction and craving management. Build self-efficacy for managing cravings and motivation for quitting.
Five	Mindful acceptance. Learn to let urges pass without smoking. Enhance self-efficacy for managing cravings and motivation for quitting. Create positive outcome expectations.
Six	Stimulus control. Learn to reduce the reinforcing effects of smoking. Enhance self-efficacy and motivation for quitting.
Seven	Cognitive restructuring. Reframe not smoking as a positive choice, not a deprivation. Support self-efficacy and create positive outcome expectations for quitting.
Eight	Successive approximation. Reduce daily smoking. Support self-efficacy and positive outcome expectations.
Nine	Put all skills into practice with a 24-hour “practice” quit. Enhance self-efficacy, motivation, and positive outcome expectations.

### Key Outcomes and Baseline Measures

#### Sources

Self-report surveys were completed on the internet at baseline, 1 month, and 3 months post enrollment. Participants received a US $25 electronic gift card for completing each survey.

App use and qualitative postexperiment ratings were assessed with automated, time-stamped data.

#### Key Outcomes of Interest

As a pilot study, we examined a range of primary and secondary outcomes to inform the feasibility, acceptability, and potential impact of the EC version relative to the SC version of GEMS. A key outcome was whether people would install the app. Among those who did, primary outcomes of interest, each assessed at 3-month follow-up, were total number of user sessions, presence of a self-reported quit attempt lasting at least 24 hours, and a self-report of no smoking for the past 7 days (7-day point prevalent abstinence [PPA]).

Secondary outcomes used to assess program use and engagement included total duration of app usage calculated as number of days between installation and last use; total number of usage badges earned; number of participants who earned enough usage badges to request free NRT; proportion of those earning NRT who requested it; the proportion of participants who clicked on the Call Now button to access free quitline counseling; and the number of people who used each app feature.

Satisfaction was assessed based on users’ overall satisfaction with their assigned program’s content and advice. All ratings used a 5-point Likert scale from “not at all” to “extremely.” In the EC arm, users also rated the helpfulness of each experiment using a 5-point Likert scale from “not helpful” to “very helpful.” Ratings were made in real time following the completion of each experiment.

Additional secondary outcomes included motivation and self-efficacy for both smoking fewer cigarettes a day and quitting smoking, each assessed as cognitive intermediaries of behavior change at the 1-month follow-up using 10-point Likert scales ranging from “not at all” to “extremely.” Other secondary indices of behavior change included a self-reported quit attempt lasting at least 24 hours, assessed at 1 month; self-reported 7-day PPA at 1 month; and the proportion of participants who reported a 50% or greater reduction in smoking from baseline to the 3-month follow-up.

Baseline assessment measures included participant demographics, use of smartphones and smoking apps, tobacco and e-cigarette use, and self-reported lifetime diagnosis or treatment for depression, anxiety, bipolar disorder, schizophrenia, alcohol use disorder, or drug use (assessed as a single yes or no for any of the listed conditions). Nicotine dependence was assessed with the Fagerström Test of Nicotine Dependence (FTND) [30]. Problem drinking was assessed with the Alcohol Use Disorders Identification Test-Consumption (AUDIT-C) [31]. Frequency of cannabis use was assessed with a single item from the Cannabis Use Disorder Identification Test-Revised (CUDIT-R) [32]. Response options for this item were never, monthly or less, 2-4 times a month, 2-3 times a week, or 4 or more times a week. Finally, we assessed participants’ outcome expectations that the help received from the study would be a key factor in either their smoking less or their quitting smoking. Each was assessed with a 5-point Likert scale ranging from 1=“strongly disagree” to 5=“strongly agree” and was modified from a similar item previously shown to predict cessation [33].

### Data and Programming Issues

Two programming issues are worth noting. First, due to a REDCap programming error, some participants who self-reported 7-day PPA at 3 months were not flagged by the system. As a result, biochemical confirmation was not obtained from these individuals as originally planned. Since these individuals made up a high proportion of individuals who self-reported 7-day PPA at 3 months, only self-reported smoking outcomes were analyzed. Second, due to a code issue, 4 EC participants were allowed to cycle through some of the experiments after a 15-minute practice period instead of the planned 24-hour period. This issue was caught early and corrected, so these individuals were retained in the analyses.

### Data Analyses

As defined a priori, outcomes are based on 2 subsets of participants. We first report on the total number of individuals who agreed to join the study and who installed the study app, as an initial indicator of study acceptability. All other analyses used a modified intent-to-treat approach and included all randomized participants who installed the app, regardless of subsequent app usage. Participants who failed to install the app were excluded from this cohort because the goal of these analyses was to assess metrics of feasibility, acceptability, and potential impact of app content among individuals who installed and used the intervention. Everyone in this analytic sample contributed automated app usage data; however, self-reported data at 1 and 3 months post enrollment were subject to missingness. Comparisons of satisfaction ratings for specific app features were restricted to participants who both self-reported use of the feature and whose automated data confirmed this use. Per convention, missing smoking outcomes were conservatively imputed as smoking. For consistency, we used a similar approach when analyzing 24-hour quit attempts (ie, missing data were imputed as not making a quit attempt). As determined a priori, secondary sensitivity analyses were also conducted for these 2 behavioral outcomes using (1) complete cases only and (2) multiple imputation by chained equation with 10 imputed data sets created with logistic regression imputation and Barnard-Rubin adjusted degrees of freedom [34,35]. For all other outcomes, analyses used complete cases without imputation.

Descriptive statistics were used to characterize the baseline sample and outcomes of interest. To compare outcomes across groups at follow-up, regression models were fitted using generalized estimating equations with robust standard errors and an exchangeable working correlation. When applicable, the model was simultaneously fitted to outcomes collected at 1 and 3 months post enrollment. For binary outcomes, we estimated relative risks (RR) of the outcome with the EC version relative to the SC version using a Poisson regression model. When events were too rare to obtain estimates of relative risks, linear regression models were used to estimate risk differences instead. Linear regression models were fitted to continuous outcomes to estimate mean differences between arms.

When applicable, to allow for separate reporting of comparisons at 1 and 3 months post baseline, time of survey collection and the interaction between follow-up time and assigned app version were included as covariates. For precision, we adjusted for the number of cigarettes smoked per day at baseline and, when applicable, baseline values of the outcome. Because groups differed at baseline by the proportion who reported a history of mental health or substance use disorder, risky drinking based on AUDIT-C scores, and household income, and these variables are known to affect cessation outcomes, we also adjusted for these potential confounders in sensitivity analyses. Point estimates are presented with 95% CIs and *P* values are from 2-sided Wald tests. All analyses were conducted in R version 4.0.2 [36].

### Sample Size

The total enrolled sample (n=60) and final analytic sample (n=57) exceed the range of 24 to 50 participants commonly recommended for pilot studies [37,38]. Smaller samples are deemed appropriate when the goal is to assess intervention feasibility and acceptability as opposed to intervention efficacy or effectiveness. The study was not powered to detect minimal clinically meaningful differences between groups with statistical significance.

## Results

### Participants

**Figure 2:**
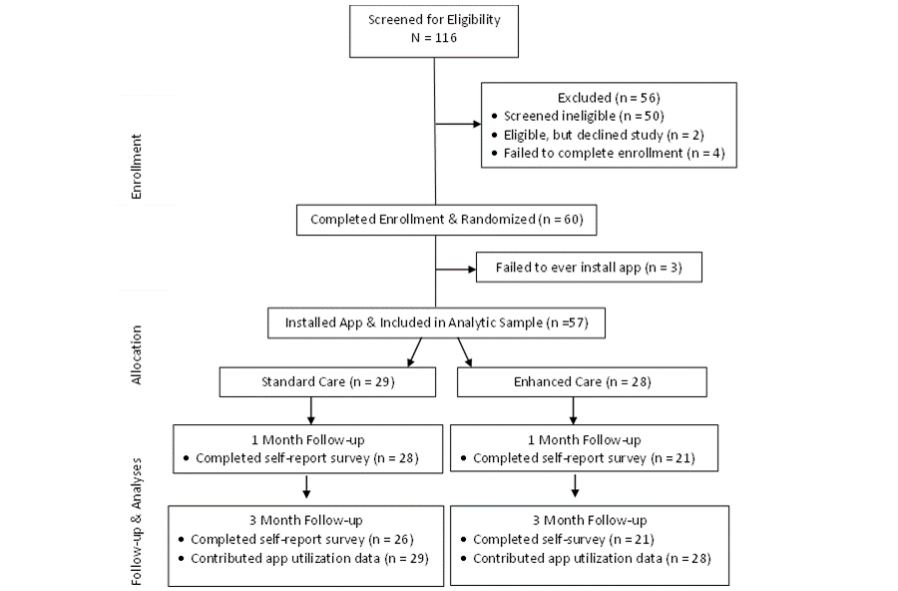
CONSORT (Consolidated Standards of Reporting Trials) flow diagram.

A total of 60 participants consented and enrolled in the study. Of these, most participants (57/60, 95%) installed the app and were included in the analytic sample (Figure 2). Demographic characteristics of this group are presented in Table 2. These participants were largely female, White, and socioeconomically disadvantaged. One-third of participants (19/57, 33.3%) had previously used health-related apps, but only 7% (4/57) had ever used a smoking cessation app. Participants smoked nearly a pack a day on average (mean 18.1 cigarettes a day) and most (36/57, 63.2%) had FTND scores indicative of “high” or “very high” nicotine dependence. Nearly one-third (18/57, 31.6%) reported using cannabis 2 or more times a week, and a similar proportion (21/57, 36.8%) self-reported previous diagnosis or treatment for either depression, anxiety, bipolar disorder, schizophrenia, alcohol use disorder, or drug use. At baseline, motivation for quitting smoking someday was moderately high (mean 6.2 out of 10, SD 1.2) and self-efficacy for quitting was moderately low (mean 4.1 out of 10, SD 1.8).

**Table 2:** Baseline descriptive characteristics.

		Overall (N=57)	Standard care (n=29)	Enhanced care (n=28)
Female, n (%)		41 (71.9)	22 (75.9)	19 (67.9)
White, n (%)		47 (82.5)	24 (82.8)	23 (82.1)
Hispanic, n (%)		0 (0)	0 (0)	0 (0)
Employed, n (%)		29 (50.9)	14 (48.3)	15 (53.6)
Annual household income <US $45,000^a^, n (%)		33 (57.9)	13 (44.8)	20 (71.4)
No college degree, n (%)		41 (71.9)	23 (79.3)	18 (64.3)
Mental health or substance use disorder (Yes)^a,b^, n (%)		21 (36.8)	9 (31)	12 (42.9)
Age (years), mean (SD)		47.5 (10.3)	47.1 (9.1)	47.9 (11.5)
Cigarettes per day, mean (SD)		18.1 (7.8)	17 (6.6)	19.2 (8.8)
FTND^c^ (nicotine dependence)^d^, mean (SD)		6.1 (1.9)	5.9 (2)	6.4 (1.7)
**Nicotine and tobacco, n (%)**				
	Use tobacco other than cigarettes (Yes)	6 (10.5)	3 (10.3)	3 (10.7)
	Use e-cigarettes (No)	47 (82.5)	23 (79.3)	24 (85.7)
	Nicotine dependence: high or very high^d^	36 (63.2)	18 (62.2)	18 (64.3)
**Substance and alcohol use, n (%)**				
	Cannabis use: 2 or more times/week^a^	18 (31.6)	9 (31)	9 (32.2)
	Hazardous drinking levels^e^	16 (28.1)	10 (34.5)	6 (21.4)
**App use, n (%)**				
	Ever downloaded health app (Yes)	19 (33.3)	10 (34.5)	9 (32.1)
	Ever downloaded smoking app (Yes)	4 (7)	2 (6.9)	2 (7.1)
**Motivation^f^, mean (SD)**				
	Reducing smoking	5.5 (1.1)	5.5 (1.1)	5.6 (1.2)
	Quitting smoking	6.2 (1.2)	6.1 (1.3)	6.2 (1)
**Self-efficacy^f^, mean (SD)**				
	Reducing smoking	3.8 (1.5)	4 (1.5)	3.5 (1.5)
	Quitting smoking	4.1 (1.8)	4.2 (1.7)	3.9 (1.9)
**Outcome expectation^g^, mean (SD)**				
	Study app will help smoke less (Yes)	3.8 (0.9)	3.8 (0.9)	3.8 (0.8)
	Study app will help quit smoking (Yes)	3.7 (0.8)	3.8 (0.9)	3.7 (0.8)

^a^Missing responses: 2 did not provide annual household income; 4 did not answer question about cannabis use; and 2 did not answer questions about mental health or substance use disorders.

^b^Self-reported diagnosis or treatment for depression, anxiety, bipolar disorder, schizophrenia, or alcohol use.

^c^FTND: Fagerström Test of Nicotine Dependence.

^d^Fagerström Test of Nicotine Dependence score. The range is from 0 to 10. Scores 6-7 indicate high dependence and 8-10 indicate very high dependence.

^e^Alcohol Use Disorders Identification Test-Consumption score. Range from 0 to 12. Scores of 4 or above for men and 3 or above for women indicative of drinking levels that are hazardous to one’s health and safety.

^f^Likert scale ranging from 1=“not at all” to 10=“extremely.”

^g^Likert scale ranging from 1=“strongly disagree” to 5=“strongly agree.”

### Indicators of Feasibility and Acceptability

#### General App Usage

A total of 3 enrolled participants failed to download the app. Among those who did install the app (57/60, 95%), usage differed significantly by group: EC participants averaged 19.9 (SD 16.2) total sessions compared to an average of 7.3 (SD 6.6) for SC users, yielding an average difference of 12.7 sessions (95% CI 6.23-18.93; *P*<.001).

Overall, duration of app usage was similar between arms but slightly favored the EC group. Mean days of use among EC users were 43 (SD 30.9) compared to 41.8 (SD 34.2) among SC users, yielding an average difference of 1.1 days (95% CI 15.65-17.85 days; *P*=.90).

Most participants (53/57, 93%) earned at least one usage badge. However, EC users earned more badges (mean 10.1, SD 6.0) than SC users (mean 6.1, SD 3.5), yielding an average difference of 4.2 badges (95% CI 1.7-6.7; *P*=.001). EC users were also more likely to earn the requisite 6 badges needed to request free NRT: 78.6% (22/28) of EC users met this bar compared to 62.1% (18/29) of SC users (RR 1.28, 95% CI 0.91-1.79; *P=*.16). Interpretation of the results was unchanged in analyses adjusting for baseline differences (data not shown).

#### Use of App Content and Features

SC and EC had similar use of the content and features common to both app versions, with 2 notable exceptions: SC users were twice as likely to indicate that they had read all of the content in the Quit Guide subsections compared to EC users, and EC users were 45% more likely to view the journal compared to SC users (see Table 3).

**Table 3:** Use of features common to both app versions.

App feature			Overall (N=57), n (%)		Standard care (n=29), n (%)		Enhanced care (n=28), n (%)		Relative risk (95% CI)^a^		*P* value^b^
**Quit Guide**											
	Step 1^c^	35 (61.4)		26 (89.7)		9 (32.1)		0.36 (0.21-0.62)		<.001	
	Step 2^c^	36 (63.2)		26 (89.7)		10 (35.7)		0.40 (0.24-0.66)		<.001	
	Step 3^c^	34 (59.6)		24 (82.8)		10 (35.7)		0.44 (0.26-0.72)		.001	
	Step 4^c^	26 (45.6)		19 (65.5)		7 (25)		0.38 (0.20-0.74)		.005	
	Step 5^c^	25 (43.9)		18 (62.1)		7 (25)		0.41 (0.20-0.81)		.01	
	Step 6^c^	25 (43.9)		18 (62.1)		7 (25)		0.41 (0.20-0.81)		.01	
Cigarette tracker^d^			24 (42.1)		13 (44.8)		11 (39.3)		0.88 (0.48-1.62)		.69
Savings calculator^e^			25 (43.9)		13 (44.8)		12 (42.9)		0.96 (0.54-1.71)		.90
Peer testimonials^f^			21 (36.8)		11 (37.9)		10 (35.7)		0.95 (0.49-1.86)		.88
Peer advice^g^			18 (31.6)		10 (34.5)		8 (28.6)		0.84 (0.39-1.80)		.65
Journal^h^			48 (84.2)		20 (69)		28 (100)		1.45 (1.14-1.85)		.003
More Help^i^			38 (66.7)		19 (65.5)		19 (67.9)		1.05 (0.73-1.49)		.80

**^a^**Relative risk (95% CI) of using that component of the app in the enhanced care arm relative to the standard care arm, adjusting for cigarettes per day at baseline. Standard care arm is the referent group.

^b^2-sided Wald test for the null of no difference in risk between arms.

^c^Based on completion of quit guide step content as defined by user marking all content in section as read.

^d^Based on use of tracker to log smoking on at least one day.

^e^Based on use of savings calculator to estimate cost savings of quitting smoking.

^f^Based on event data showing each peer testimonial modeling how to talk back to common excuses for not quitting was opened and viewed.

^g^Based on event data showing each vignette providing motivational support and advice was opened and viewed.

^h^Based on event data showing the Journal was opened at least one time, whether or not an entry was created.

^i^Based on opening the More Help page at least one time to access tobacco quitline referral and other information on where to get help quitting smoking.

#### Overall Satisfaction Ratings

Self-reported satisfaction with participants’ assigned app version was similar in both groups. At 3-month follow-up, most respondents reported they would recommend the app to others (19/20, 95% EC users vs 22/23, 95.7% SC users; RR 1.03, 95% CI 0.89-1.19; *P=*.70). Among respondents who earned at least one usage badge by 3 months post enrollment (n=43)—the minimum exposure threshold deemed adequate to evaluate the app content—respondents in both arms reported similarly high satisfaction with their assigned app’s overall content and advice. Mean satisfaction ratings in both arms were 4.1 out of 5 (SD 1.1). Similar results were observed at 1-month follow-up; mean satisfaction ratings were 3.6 (SD 1.2) among SC users and 3.8 (SD 1) among EC users (adjusted average difference 0.26, 95% CI –0.35 to 0.87; *P=*.40).

#### Experiment Engagement and Helpfulness

EC users completed an average of 6.14 (SD 3.31) and 6.89 (SD 3.08) experiments by 1- and 3-month follow-up, respectively. Completion rates across each of the 9 individual experiments ranged from 93% (26/28) to 61% (17/28) (Table 4).

Immediately after completing each experiment, EC users were asked to rate the helpfulness of the experiment. Median helpfulness scores ranged from 3 to 4 on a 5-point Likert scale (Table 4). Experiments receiving the highest median scores focused on learning to identify high-risk situations and triggers for smoking, reducing daily smoking, and making a practice quit attempt (median 4 for each). Exercises focused on learning deep breathing for stress reduction and reframing not smoking as a personal choice (as opposed to a deprivation) also received higher median scores (3.75).

**Table 4:** Portion of enhanced care participants completing each experiment and median helpfulness ratings.

Experiment	At 1-month follow-up^a^, n (%)	At 3-month follow-up^a^, n (%)	Helpfulness^b^, median (IQR)	Range (minimum-maximum)^b^
One	25 (89)	26 (93)	3 (3-4)	1-5
Two	22 (79)	23 (82)	3 (2-4)	2-5
Three	21 (75)	23 (82)	4 (3-4.25)	1-5
Four	20 (71)	22 (79)	3.75 (2.25-4)	1-5
Five	20 (71)	22 (79)	3 (2.25-4)	1-5
Six	19 (68)	22 (79)	3.5 (3-4.75)	1-5
Seven	18 (64)	20 (71)	3.75 (2-4.08)	1-5
Eight	15 (54)	18 (64)	4 (3-4.83)	3-5
Nine	12 (43)	17 (61)	4 (3-5)	2-5

^a^The number and proportion of all enhanced care participants (n=28) who completed each experiment by 1- and 3-month postenrollment follow-up.

^b^Reflects the median (IQR), and range (minimum-maximum) of helpfulness ratings across all experimental participants who completed each in-app, postexperiment assessment. If a user completed the experiment more than once, the average of their ratings was used. Ratings could range from 1=“not at all” to 5=“extremely helpful.”

### Indicators of Intermediate Cognitive Change at 1 Month

#### Motivation

Among participants still smoking at 1-month follow-up, mean self-reported motivation to quit was 8.2 (SD 2.1) for EC users compared to 7.1 (SD 2.6) for SC users (adjusted mean difference 0.54, 95% CI –0.49 to 1.57; *P*=.30). Motivation to smoke less at the 1-month follow-up averaged 8.3 (SD 1.7) for EC users compared to 7.4 (SD 2.5) among SC users (adjusted mean difference 0.92, 95% CI –0.07 to 1.92; *P*=.07). In both groups, indices of motivation increased from baseline (Table 2) to follow-up.

#### Self-Efficacy

Among participants still smoking at 1-month follow-up, mean self-efficacy for quitting smoking was 7.4 (SD 2.4) for EC users compared to 7.5 (SD 2.3) for SC users (adjusted mean difference 0.03, 95% CI –1.02 to 1.07; *P*=.96). Self-efficacy for smoking less at the 1-month follow-up averaged 6.3 (SD 2.2) for EC users compared to 7.7 (SD 2.5) for SC users (adjusted mean difference 0.06, 95% CI –0.97 to 1.09; *P*=.06). In both groups, indices of self-efficacy increased from baseline (Table 2) to follow-up.

### Indicators of Behavior Change at 3 Months

#### Requests for Free NRT

Among the 40 participants who earned 6 usage badges and were eligible to request a free trial of NRT, 10 participants requested it (8/22, 36.4% EC users as compared to 2/18, 11.1% SC users; RR 3.18, 95% CI 0.77-13.17; *P=*.11).

#### Call Now for Free Counseling

At the 3-month follow-up, 17.9% (5/28) of EC participants and 3.4% (1/29) of SC participants had clicked on the Call Now button to connect with a free tobacco quitline counselor (RR 5.17, 95% CI 0.65-41.3; *P*=.12).

#### Smoking Reduction

At the 3-month follow-up, a similar proportion of participants using both app versions reported a significant reduction in their daily smoking rate: 28.6% (6/21) of EC users compared to 28% (7/25) of SC users (RR 0.01, 95% CI –0.25 to 0.27; *P*=.92) reported a 50% or greater reduction in their baseline daily smoking.

#### Quit Attempts

At the 3-month follow-up, 39.3% (11/28) of EC users and 37.9% (11/29) of SC users reported making an intentional quit attempt after joining the study, when missing values were imputed as not making a quit attempt (RR 1.01, 95% CI 0.55-1.85; *P*=.98). The interpretation of the results was unchanged in complete case and multiple imputation sensitivity analyses or analyses adjusting for baseline differences (results not shown).

#### Smoking Abstinence

At the 3-month follow-up, 14.7% (4/28) of EC users and 6.9% (2/29) of SC users reported not smoking, even a puff, in the last 7 days (risk difference 0.08, 95% CI –0.08 to 0.24; *P=*.35). The interpretation of the results was unchanged in complete case and multiple imputation sensitivity analyses or analyses adjusting for baseline differences (data not shown).

## Discussion

### Principal Findings

The primary objective of this randomized pilot study was to evaluate the feasibility and acceptability of the EC version of the GEMS app and to assess its potential to motivate and support smoking behavior change compared to a similar app that included SC content (SC version) but was not designed specifically for smokers who are ambivalent about quitting smoking. It is encouraging that 95% of the participants who agreed to enroll in the study installed the app. This provides an important initial signal of the intervention’s acceptability. However, because of the size and nature of the study, conclusions about the acceptability and impact of the intervention among participants who installed the app cannot be based on the statistical significance of the primary and secondary outcome comparisons. Instead, it is important to look at the trend and pattern of the point estimates at follow-up. Notably, in almost all cases, the observed outcomes trended in favor of the EC app version. This held true for both self-reported outcomes and those based on objective automated data.

As hypothesized, EC participants used the app more often (an average of 19.9 sessions vs 7.3 sessions for SC participants), and a greater proportion reported smoking abstinence at follow-up (14.7% of EC participants vs 6.9% of SC participants). This finding is consistent with previous research showing an association between greater program engagement, or adherence, and higher cessation rates [39,40]. The observed quit rate in the EC arm is similar to that observed from physician advice to quit and low-intensity counseling interventions (average 14%-16%) [9].

Additionally, EC participants earned an average of 4 more usage badges. Notably, EC participants had the potential to earn more badges based on the additional content (experiments) in this version, but both groups had equal opportunity to earn the requisite 6 badges needed to request a free trial of NRT, and a higher proportion of EC users met this bar based on their app usage (78.6% EC users vs 62.1% SC users). Additionally, more EC users who earned the NRT, requested to receive it (36.4% EC vs 11.1% SC). Similarly, at the 1-month follow-up, motivation for quitting smoking trended higher in the EC group (8.3 EC vs 7.4 SC on a 10-point scale), even though groups had similar self-efficacy for quitting. More EC users clicked the Call Now button in the app to access free quitline counseling (17.9% of EC vs 3.4% of SC), although it is not known if these individuals actually enrolled in the free quitline program. Finally, a slightly higher proportion of EC users (39.3%) reported making a quit attempt compared to SC users (37.9%) and 28.6% of EC users reported a meaningful reduction in daily smoking at the 3-month follow-up.

It is also notable that participants in this trial were lower–socioeconomic status heavy smokers, with high levels of concomitant substance use or lifetime substance-related or mental health diagnoses. These groups have been shown to be less likely to engage in treatment and successfully quit smoking [41,42] and, therefore, represent important targets for intervention.

Taken together, these findings confirm the conclusions drawn from our previous formative work that smokers who are ambivalent about quitting, including those who are more socioeconomically disadvantaged, are interested in using an mHealth app to help them reduce or stop smoking [17-19] and that designing this intervention to be sensitive to participants’ ambivalence about quitting could increase their likelihood of changing their smoking behavior.

### Role of the Experiments

A key question in understanding the feasibility of this app was whether ambivalent smokers would engage with the self-directed, smoking-focused, cognitive, and behavioral experiments if they were not yet ready to commit to quitting smoking. The experiments were designed to teach users specific skills and lessons to help people resist the urge to smoke and encourage a quit attempt. These skills are consistent with the common elements of effective behavioral interventions identified in the US Public Health Service’s Tobacco Treatment Guidelines (ie, problem-solving skills, such as identifying high-risk situations, coping skills for managing urges without smoking, basic educational information, and supportive encouragement to make a quit attempt) [9]. Inclusion of these elements has been shown to increase the effectiveness of low-intensity counseling. The results of this pilot suggest these elements may also be useful in self-directed mHealth interventions, though we also acknowledge the importance of the EC reminder prompts. As hypothesized based on Fogg’s behavioral model for persuasive design, these prompts appear to have aided continued program engagement [29].

Findings from this study also indicate that most EC participants were willing to try the exercises. Completion rates ranged from 93% of EC users completing the first experiment (clarifying one’s values) to 61% completing the last experiment (making a practice quit attempt). These rates are encouraging. Because the addition of the experiments was the key content difference between the 2 app versions, engagement with the exercises likely drove the favorable trends in the outcomes noted above. This is further supported by the fact that EC participants were less likely than SC participants to read the smoking cessation Quit Guide. This was likely an artifact of the differences in the positioning of this content in the app (it was on the home page in the SC app version and located in the Toolbox linked from the home page in the EC app version), but because EC participants were less likely to view this content, exposure to the Quit Guide cannot explain the more favorable outcomes observed in the EC arm.

The graphical user interface for accessing the Quit Guide does not appear to have been an impediment to app usage or behavior change in the EC arm, but it is worth considering if the Quit Guide should be featured more prominently in a future EC version and, if so, whether this would add additional value to users or not.

### Limitations and Strengths

The findings from this work must be viewed in the context of the study limitations. Chief among these is the small sample size; the study was not powered to detect minimal clinically meaningful differences with statistical significance, and the sample size limits our ability to draw any firm conclusions about the generalizability of the findings, particularly with regard to smoking behavior change. Additionally, cessation outcomes are based on self-reported data and are subject to social desirability bias. However, biochemical confirmation is not generally recommended in trials with no face-to-face contact and where the demand characteristics to misreport abstinence are low [43], such as this remote trial of smokers who are ambivalent about quitting smoking. Moreover, relying on remote biochemical confirmation of smoking abstinence has been shown to bias outcomes due to low rates of participation [44]. For these reasons, the use of self-reported cessation outcomes is reasonable for this preliminary work. However, self-reporting also has its limitations. In this study, we saw a higher rate of attrition at the 3-month follow-up in the EC arm, resulting in a higher number of imputed smokers in this arm. Despite this, cessation outcomes still favored the EC group.

To our knowledge, this is the first app to have been designed specifically for smokers who are ambivalent about quitting, thus addressing an important intervention gap. Other strengths include the recruitment of a high-risk and low–socioeconomic status sample, a rigorous methodological design that allows the unique effects of the experiments to be tested, reliance on both self-report and automated tracking data for outcomes, and overall strong follow-up participation at 3 months (47/57, 82.5%).

### Conclusions

This study provides encouraging evidence that people who are ambivalent about quitting smoking will voluntarily use and remain engaged with an mHealth app that is designed to help them cut back or quit smoking, even if they are not actively planning to change their smoking behavior at program initiation. Further development of app-based interventions targeted at smokers who are ambivalent about quitting is warranted, as is further evaluation of the effectiveness of EC app version.

## Appendix

Multimedia Appendix 1 CONSORT eHEALTH checklist (V 1.6.1).

## Abbreviations

AUDIT-C: Alcohol Use Disorders Identification Test
CUDIT-R: Cannabis Use Disorder Identification Test-Revised
EC: enhanced care
FTND: Fagerström Test of Nicotine Dependence
KPWA: Kaiser Permanente Washington
mHealth: mobile health
NRT: nicotine replacement therapy
PPA: point prevalent abstinence
RR: relative risk
SC: standard care

## Data Availability

This pilot work was not subject to the National Institutes of Health data sharing requirements and did not obtain consent or institutional review board approval for publicly sharing data. As such, the data sets analyzed for this study are not publicly available, but we will consider sharing data upon request and with institutional review board approval.
